# Wake Development behind Paired Wings with Tip and Root Trailing Vortices: Consequences for Animal Flight Force Estimates

**DOI:** 10.1371/journal.pone.0091040

**Published:** 2014-03-14

**Authors:** Jan T. Horstmann, Per Henningsson, Adrian L. R. Thomas, Richard J. Bomphrey

**Affiliations:** 1 Bremen University of Applied Sciences, Bremen, Germany; 2 Department of Zoology, University of Oxford, Oxford, United Kingdom; 3 Department of Biology, Lund University, Lund, Sweden; 4 Structure & Motion Laboratory, The Royal Veterinary College, London, United Kingdom; University of Zurich, Switzerland

## Abstract

Recent experiments on flapping flight in animals have shown that a variety of unrelated species shed a wake behind left and right wings consisting of both tip and root vortices. Here we present an investigation using Particle Image Velocimetry (PIV) of the behaviour and interaction of trailing vortices shed by paired, fixed wings that simplify and mimic the wake of a flying animal with a non-lifting body. We measured flow velocities at five positions downstream of two adjacent NACA 0012 aerofoils and systematically varied aspect ratio, the gap between the wings (corresponding to the width of a non-lifting body), angle of attack, and the Reynolds number. The range of aspect ratios and Reynolds number where chosen to be relevant to natural fliers and swimmers, and insect flight in particular. We show that the wake behind the paired wings deformed as a consequence of the induced flow distribution such that the wingtip vortices convected downwards while the root vortices twist around each other. Vortex interaction and wake deformation became more pronounced further downstream of the wing, so the positioning of PIV measurement planes in experiments on flying animals has an important effect on subsequent force estimates due to rotating induced flow vectors. Wake deformation was most severe behind wings with lower aspect ratios and when the distance between the wings was small, suggesting that animals that match this description constitute high-risk groups in terms of measurement error. Our results, therefore, have significant implications for experimental design where wake measurements are used to estimate forces generated in animal flight. In particular, the downstream distance of the measurement plane should be minimised, notwithstanding the animal welfare constraints when measuring the wake behind flying animals.

## Introduction

Vortex structures in the wake of aerial and aquatic animals can be interpreted as a historical representation of the forces generated during locomotion. According to Newton’s second and third laws, these structures contain time-integrated information about the forces produced by the animal in order to propel itself through the fluid. Particle Image Velocimetry (PIV) provides a useful method to extract this information from the wake by measuring velocity vector planes through the flow field [1]–[6]. Analysed with an appropriate model, these data can yield estimates of the forces generated by the animal.

Biologist and engineers interested in the mechanics of swimming and flying commonly use two models to analyse measured flow fields: the *vortex ring model* and the *circulation model*. The vortex ring model was originally developed for the investigation of bird flight [7], [8], but has also become a powerful tool in aquatic propulsion studies [9]–[12]. The model is formulated on the assumption that the entire energy shed into the wake concentrates into vortex rings of circular or elliptical shape. The impulse of a single vortex ring is calculated as.
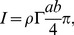
(1)where *a* is the diameter in the *x*-direction and *b* is the diameter in the *y*-direction of the vortex ring, ρ is the density of the fluid and Γ is the circulation of the vortex ring, which is either computed as the line integral of velocity, *V*, around the vortex core or the area integral of vorticity, ω:



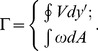
(2)The force, *F,* is then defined as the impulse, *I,* over time, where τ is the duration of the vortex ring generating motion (such as the half wingbeat cycle of a flapping insect or the half tailbeat cycle of an undulatory swimming fish). Thus it represents a time-averaged estimate of the force,
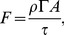
(3)where 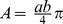 is the area of the vortex ring.

The *circulateon model*, in contrast, relates the circulation measured in a single image frame to the instantaneous force production. This relation, from the Kutta–Joukowski theorem, is a useful tool for lift calculation in animal flight studies:
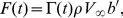
(4)where Γ is the circulation in the trailing vortices, *V*_∞_ the free stream velocity and *b*′ the wake span. Time-resolved PIV, where multiple images of the wake can be captured within a single stroke cycle, allows for estimates to be made of the magnitude, timing and direction of the force during the progression of a wingbeat [13].

A common assumption of animal flight models is that the wake topology is preserved as it convects downstream with the freestream velocity *V*_∞_. In the Lagrangian frame of reference, the wake topology is time-independent, and is referred to by Taylor as the frozen flow hypothesis [3], [14], [15]. Following this assumption, the wake structure related to a certain event during the stroke cycle, such as supination or pronation, can be found in the wake by following the transformation *x* = *V*_∞_/Δ*t*, where *x* is the downstream distance behind the foil and Δ*t* is the time that has passed since the structure was generated.

Similarly, the vortex ring model applied to aquatic locomotion requires the recording of at least one vortex loop. The distance between the recorded vortex structure and the appendage is normalized by the span (*x*/*s,* where *s* is the fin span). Depending on the swimming velocity and the duration of the half stroke (*x* = *V*×τ) the loop can have a horizontal dimension of up to *x*/*s* ≈ 2, as has been recorded from the wake of a rainbow trout *Oncorhynchus mykiss* [12]. In this example, the visualization of a vortex ring recorded immediately after shedding captures a structure in which the distal part of the ring has convected approximately two span lengths prior to measurement. The convention in animal locomotion research of assuming the Taylor hypothesis may have led to the potential effect of measurement plane position being largely neglected–especially in wind tunnel work–and, consequently, the measurement position has varied considerably between experiments. Normalized by the mean chord length, *c*, measurement plane downstream distances range from directly on the wing (*x*/*c* ≈ 0, desert locust *Schistocerca gregaria* [1],[2] and Palla’s long-tongued bat *Glossophaga soricina* [16]) to intermediate downstream distances (*x/c* ≈ 10, common swift *Apus apus* [4] and blackcap *Sylvia atricapilla* [17]) to far wake measurements (*x*/*c* ≈ 20, Lesser short-nosed fruit bat *Cynopterus brachyotis* [5]).

Wake deformation behind a flying insect was recently described using high-speed, tomographic PIV and deformation of the complex wake was found to be pronounced even over a relatively short distance and timescale (∼1 chord) [13]. Deformation becomes challenging when reconstructing the three-dimensional wake both when using planar and large-volume PIV because of a potential misrepresentation of the wake shape and orientation: in planar PIV because of the spatio-temporal separation between the sampled vector fields, and in large-volume PIV because of the varying ages of the wake elements captured within the same volume (where elements closer to the animal were shed more recently than those further away [5]). It has been demonstrated that these constraints could partially be improved using dual plane PIV [18].

### Trailing Vortex Interaction

**Figure 1:**
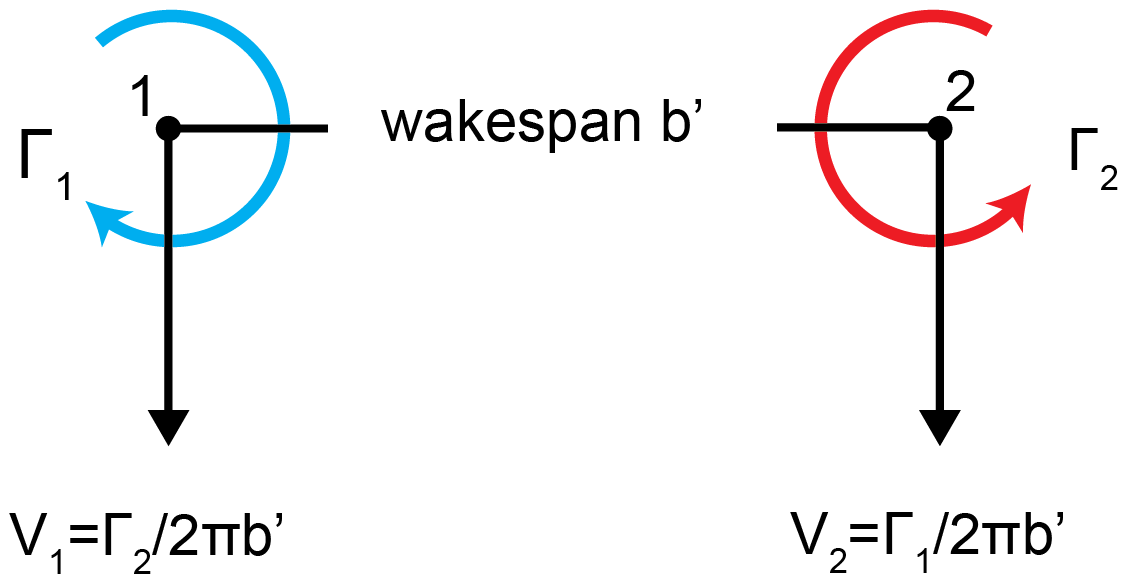
A pair of counter rotating trailing vortices: At any position outside its core a vortex induces a velocity, which equals the circulation Γ divided by the circumference of the vortex at that position 2π*r* (free vortex). In a pair of trailing vortices the mutually induced velocities are therefore calculated for *r* equals *b*’, which is the displacement between the vortices. In this case the induced velocities each vortex experiences are directed downwards.

Known far-wake vortex interaction phenomena, such as the Crow instability [19], have been neglected in studies that use the animal’s signature in the wake to estimate forces. In most instances this is reasonable, since instability develops at downstream distances exceeding typical measurement plane locations. However, vortices impose an induced velocity field that can also lead to mutual interaction effects between closely spaced vortices in the near-wake [20]. The tangential or induced velocity field as a function of the radius, *r*, can be predicted using the Rankine vortex model,
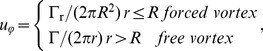
(5)where *r ≤ R* corresponds to the forced vortex core and *r*>*R* corresponds to the free vortex. For the purpose of this study, we are principally interested in the tangential velocity outside the core–beyond the region of solid body rotation–which is the circulation, Γ, of the vortex divided by its circumference (2π*r*) and thus decreases hyperbolically with distance from the vortex centre. The effect of a mutually induced velocity field of two vortices is best demonstrated by the wake of a lift-generating wing (Fig. 1). Provided that the magnitude of the circulation in the two counter-rotating trailing vortices is the same, the system translates downwards at a velocity *V* = Γ/2π*b*′ (where *b*′ is the wake span), whilst simultaneously being transported downstream with the free stream.

For many years it has been assumed that the same vortex system captures the characteristics of the wake generated by birds, bats and insects in the vertical transverse plane. The calculation of lift force using the Kutta-Joukowsky theorem is in such case not affected by the downward translation of the system because the wake span, *b*′, remains unaffected. However, recent flow visualizations in the vertical transverse plane of birds, bats [4], [5], [17], [21] and insects [22], [23] have revealed a complex wake topology that may reduce the efficacy of simplified models. In addition to the two wing tip vortices, a pair of wing root vortices appears to be a common feature that has recently been discovered in birds, bats and insects thanks to improvements of the PIV technique resulting in higher temporal and spatial resolution (e.g. [24]; c.f. [5], [17], [22], [25]). Due to their proximity to one another and their opposite sense of rotation, these vortices are likely to interact and thereby alter the wake morphology and any subsequent calculations that rely on that morphology. If so, the frozen flow assumptions are not valid and error may be introduced into the direction, magnitude and timing of force estimates. Consequently, the aerodynamic models would have to be modified in order to account for time-dependent wake effects. Thus, it is important to know which features of the wings and their configuration that have an impact on wake deformation, and how significant that impact will be. We used a simplified fixed-wing model to generate a wake consisting of two wing tip and two wing root vortices to explore vortex interaction behind wings with geometry and flow conditions relevant to animal flight experiments.

## Materials and Methods

### The Paired-wing Apparatus

**Figure 2:**
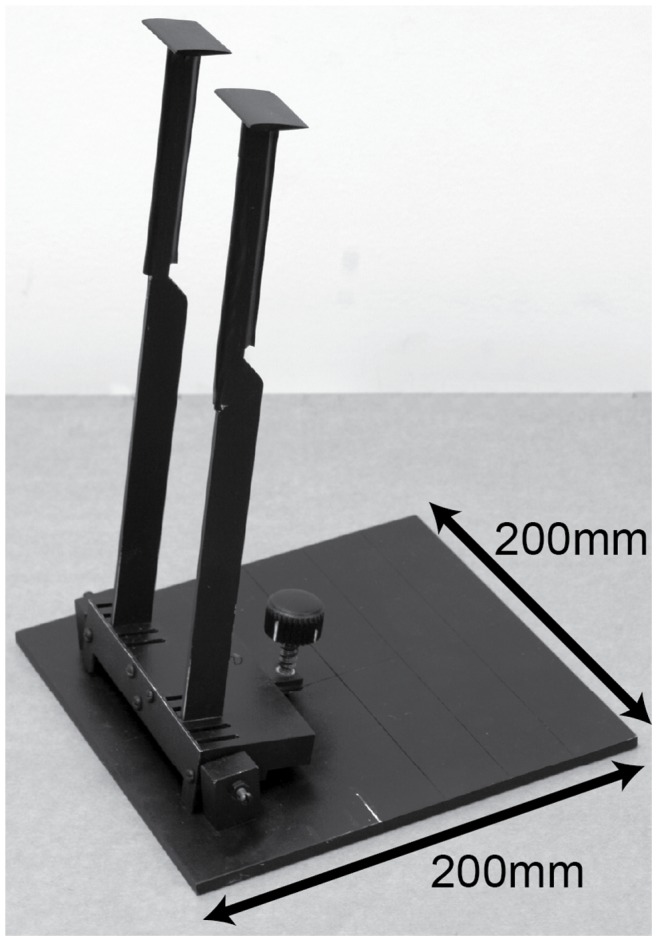
The paired wing apparatus. A pair of NACA 0012 wings was mounted on two tapered aluminium supports, which inserted into tight-fitting slots of variable displacement in a plastic frame. Attached to the base plate via a hinge, the angle of attack of the frame was adjustable by a rearward positioned screw.

Two sets of rectangular NACA 0012 wings with chord length 24 mm and aspect ratios of 1, 1.5 and 2 were constructed using a 3D-printer (Zprinter450, ZCorporation, Burlington, USA). The surface of the wings were smoothed with fine sandpaper after hardening and painted matt black. Matching pairs of wings were attached to aluminium supports of 270 mm length. The narrower, upper half of the support was 8×3 mm in cross-section and the lower half (18×3 mm) inserted into a row of slots milled into a plastic frame. An adjustable screw gave quick, fine adjustment of the articulated base plate. This design allowed us to change the angle of attack, the aspect ratio, and the gap separating the wing pair, which simulates varying width of a non-lifting body surface between the wings (Fig. 2).

### PIV Setup

Experiments were conducted in an open-return, low-turbulence wind tunnel with a contraction ratio of 16∶1 [26]. We used a high-speed, stereo PIV system with DaVis 7.2.2 control, acquisition and processing software (LaVision UK Ltd, UK). The flow was seeded with olive oil particles (∼1 μm) produced with a compressed air aerosol generator (LaVision UK Ltd, UK) at the wind tunnel intake. Seeding particles were illuminated with a double-pulsed 10 mJ laser (Litron LDY-300PIV, Nd-YLF, 527 nm, Litron Lasers Ltd, UK) at a double-pulse rate of 1 kHz. The beam was spread into a vertical transverse plane (c. 2 mm width), normal to the freestream, by a −10 mm cylindrical lens and delivered into the test section from above. Two high-speed CMOS-sensor cameras (Photron SA3∶1024×1024 px; 2000 fps) with 50 mm lenses (AF Nikkor, f/1.9), mounted on Scheimpflug adapters (LaVision UK Ltd, UK), were placed at either side of the test section. The overlapping field of view of the two cameras was approximately 120×120 mm. Interrogation windows of 32×32 px were cross-correlated with a standard fast Fourier transformation algorithm. For each wing-configuration 250 image files were averaged to give a single vector field corresponding to 0.25 s. The average error in our PIV-setup has been described elsewhere by calculating the RMS across 100 vector fields of freestream flow and found to be 0.20 m s^−1^ [27]. This can be viewed as an estimate of the average error of the velocities–grouping together the effect of spatial variation in velocities across the test section, wind tunnel turbulence and PIV vector calculation errors.

### Experimental Protocol

The paired wing apparatus allowed for varying three parameters: angle of attack, aspect ratio and the horizontal spacing between the wings. In addition we varied the distance between the trailing edges of the wings and the laser sheet by translating the mount upstream. Finally, we varied the Reynolds number by changing the freestream velocity. An overview of the parameter space is given in Table 1.

**Table 1:** Summary of parameter space.

Parameter	Value	*n*
Reynolds number	4.5×10^3^; 6.0×10^3^; 7.5×10^3^	3
Angle of attack	5°; 8°	2
Wing aspect ratio	1; 1.5; 2	3
Gap normalised by chord length	1/3; 2/3; 3/3	3
Downstream distance normalised by chorld length (*x*/*c*)	1; 2; 5; 10; 17	5
		*Total combinations*: 270

### Analysis

**Figure 3:**
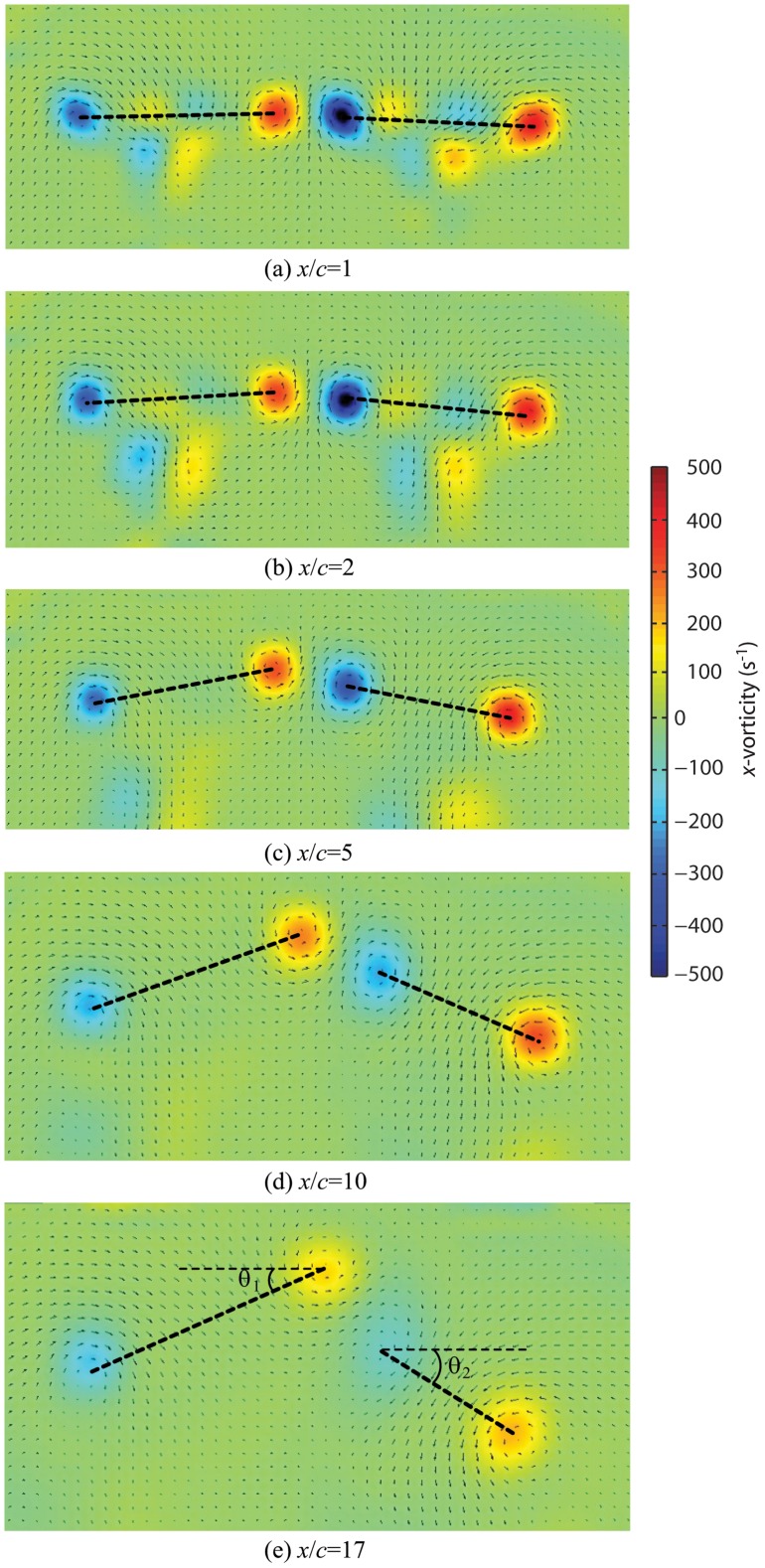
Vortex interaction in the wake of two paired NACA 0012 wings at *Re* = 6000. Angle of attack, 5°; single wing aspect ratio, 1.5; gap width, 1/3 chords. The normalized downstream distance is increased from 1 to 17 chord-lengths. Bottom panel (E) shows how the wake angles were defined.



The time-averaged flow field vector files were loaded into a custom written MATLAB analysing tool in order to digitize manually the coordinates of the centre of the vortices in the flow fields. Defining the vortex cores as the centre of rotation around the freestream axis, determined with respect to the strength and orientation of the nearby velocity vectors, each averaged vector field was used to extract coordinates for the two wingtip and two wing root vortex cores. Deviation from a horizontal arrangement of the trailing vortices was deemed the wake rotation angle, defined as the mean absolute angle, 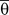, that the two transects running through the counter-rotating vortex pairs from each wing made with the horizontal (Fig. 3E). The angles θ_1_ and θ_2_ between the two transects were calculated as:
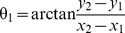
(6)


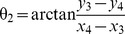
(7)where *x*_i_ and *y*_i_ (*i* = {1, 2, 3, 4}) are the horizontal and vertical coordinate directions of the four trailing vortices in the vertical transverse plane.

### Statistical Model

Statistical analyses were performed using in PASW Statistics 18 (SPSS Inc., Chicago, IL, USA). We investigated the relationship between the wake angle and the parameters given in Table 1 using a general linear model with a multi-factor ANOVA design with the wake angle as the dependent variable and the Reynolds number, the angle of attack, the aspect ratio, the gap between the wings, and the normalized downstream distance (*x*/*c*) as independent variables. Both positive and negative relationships were analysed (i.e. two-tailed test) and deemed significant if *p*<0.05.

## Results

Figure 3 shows a typical set of PIV recordings captured in the vertical transverse plane at increasing downstream distances in the wake behind the paired wing configuration. At one chord length behind the trailing edges (*x*/*c* = 1; Fig. 3A), four distinct vortices with alternating sense of rotation can be recognized. For the sake of analogy with animal flight experiments, the two outer vortices will be referred to as tip vortices and the two inner as root vortices. With increasing downstream distance, the trailing vortices become more diffuse (Fig. 3B–E). Moreover, they begin to shift, with the tip vortices convecting downwards while the root vortices remain at their vertical level but ultimately twist around each other (Fig. 3D–E).

**Figure 4:**
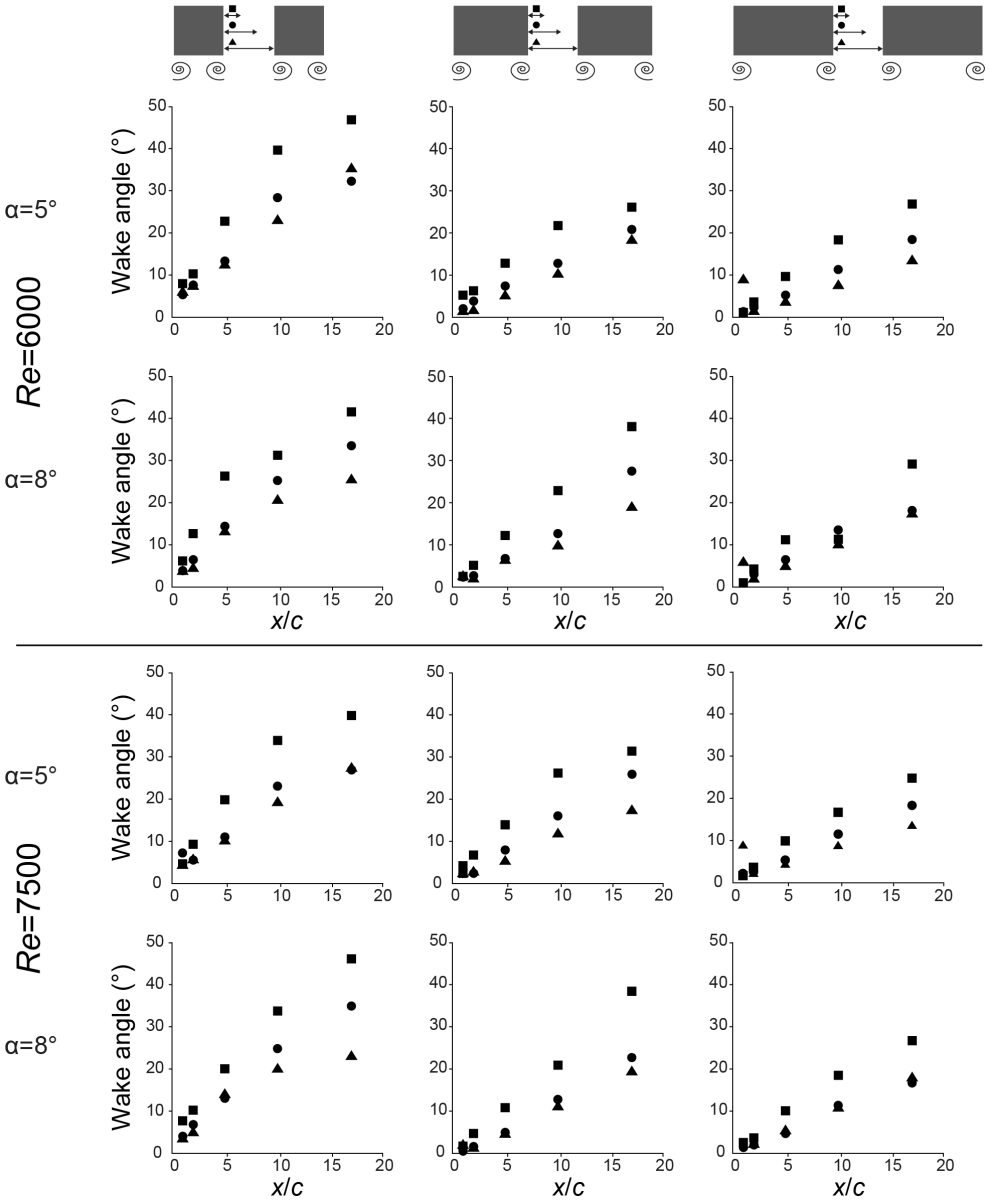
Scatter plots of the measured wake angle. Reynolds number 6000 and 7500 are presented as the upper and lower half respectively. Rows show the two different wing angles of attack at the two Re and columns show different wing aspect ratios. Squares show the smallest gap (1/3 chord length), circles show the medium gap (2/3 chord lengths) and triangles show the largest gap (1 chord length).

The measured wake angles for different gap distances and at different downstream distances are presented in Figure 4 for the two Reynolds number and the three aspect ratios. At lower aspect ratios and as the gap between paired wings becomes smaller, wake deformation becomes more pronounced (aspect ratio: *B* = −9.14, *t* = −8.83, *p*<0.001, gap: *B* = −0.67, *t* = −7.52, *p*<0.001). As expected, wake deformation also becomes increasingly pronounced as the measurement plane is moved further away from the wings (*B* = 3.60, *t* = 28.43, *p*<0.001). Higher velocities, and therefore Reynolds number, result in a smaller deformation of the wake although the relationship is weaker than the other effects (*B* = −4.60×10^−4^, *t* = −2.94, *p*<0.001). The angle of attack of the wings, which is expected to correlate with circulation and lift at small angles, has no significant effect over the range tested in our experiment (*B = *−0.002, *t* = −0.018, *p* = 0.99).

Aspect ratio has the largest impact on wake deformation within our parameter space. Increasing the ratio of span over chord by one, leads to an average reduction in the wake angle by more than 9° when taking all downstream distances into account (*B* = −9.14). Less pronounced, but still considerable, is the effect of the distance between the trailing edge and the measurement plane. Increasing the distance by one chord length increases the wake angle by 3.6° (*B* = 3.60). Changing the gap width and the velocity by one unit has little effect on the wake deformation. In both cases the resulting deformation is less than one degree (gap: *B* = −0.67; Re: *B* = −4.60×10^−4^) when taking all downstream distances into account.

We also found significant interaction effects between the aspect ratio and downstream distance (*B* = −0.85, *t* = −12.52, *p*<0.001), the gap width and the downstream distance (*B* = −0.051, *t* = −11.80, *p*<0.001) and the aspect ratio and the gap width (*B* = 0.33, *t* = 5.99, *p*<0.001). As shown by the *B*-values, the influence on the wake deformation with respect to these interactions is comparatively small.

In summary, four of the five parameters we tested, had a significant effect on the wake deformation with aspect ratio and downstream measurement distance being the most important factors (Table 2).

**Table 2:** Summary of the statistical test with upper and lower bounds based on 95% confidence intervals.

Parameter	*B*	*t*	*p*	Lower	Upper
				Bound	Bound
Intercept	21.593	10.445	<0.001	17.528	25.658
Reynolds number	−4.60×10^−4^	−2.941	0.003	−7.67×10^−4^	−1.52×10^−4^
Angle of attack	−0.002	−.018	0.986	−0.213	0.209
Aspect ratio (AR)	−9.136	−8.829	<0.001	−11.171	−7.101
Gap	−0.672	−7.517	<0.001	−0.848	−0.496
Distance	3.597	28.429	<0.001	3.348	3.846
AR×Gap	0.33	5.988	<0.001	0.221	0.438
AR×Distance	−0.850	−12.524	<0.001	−0.983	−0.717
Gap×Distance	−0.051	−11.796	<0.001	−0.059	−0.042

## Discussion

### Physical Explanation of the Wake Deformation

**Figure 5:**
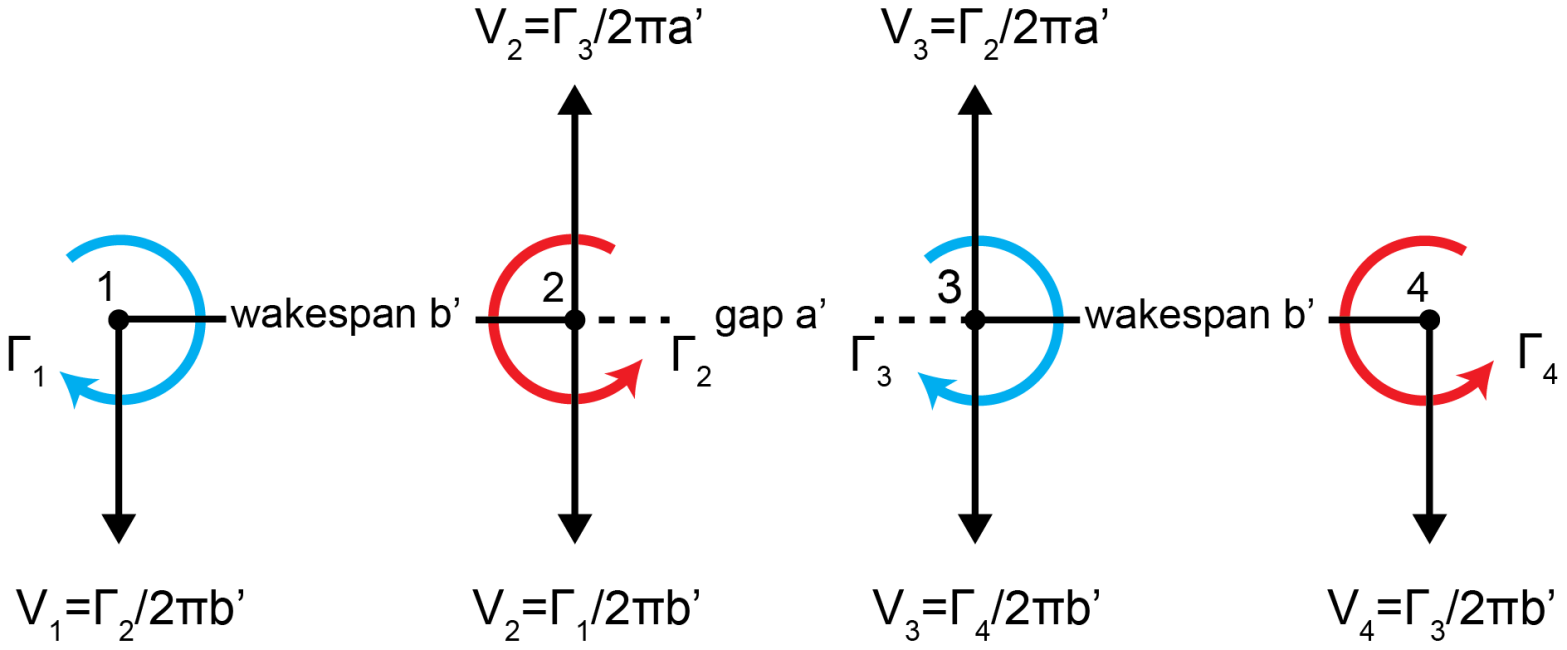
Diagram of two pairs of counter rotating vortices. As in Figure 1, induced velocities are calculated for *r* equals *a*’ and *b*’, which are the displacements between the vortices. The major difference from a single vortex pair system is the bi-lateral impact of the induced velocities. Vortex 2 experiences downwards directed velocity induced by vortex 1 and upwards directed velocity induced by vortex 3. If these velocities are of same magnitude, vortex 2 will experience no net displacement.

The wake, consisting of two pairs of counter-rotating vortices, deforms with time. In particular the tip vortices exhibit a downward motion, whereas the root vortices largely maintain their vertical position but twist around each other. One explanation is the mutual interaction of the vortices [28]. In Section 2 we considered a conventional single wing wake-system, consisting of two trailing vortices of same strength Γ and opposite sense of rotation. We concluded that due to the mutually induced velocities a vortex pair travels downwards at a velocity of *V* = Γ/2π*b’* as it convects downstream with the flow (Fig. 1). Considering another such system adjacent to the first, the root vortices can be expected to interact when their displacement is on the order of the wake span *b*’ (Fig. 5). Their mutually imposed velocities are directed upwards, which balances the downwards directed velocity induced by the presence of the tip vortices. Consequently, the displacement between the two root vortices, and their circulation, affects their vertical translational motion within the wake and the resulting wake angle. If the gap equals the wingspan and the circulation is constant among the vortices, the induced velocities on the root vortices are balanced, while the tip vortices experience a downward directed velocity. This vortex interaction behaviour, which we were able to demonstrate in our experiments, and for which there is a simple explanation, challenges the frozen flow hypothesis in the context of aerial and aquatic locomotion.

### The Influence of Root Vortices on Conventional Wake Measurements Methods

Root vortices are a wake feature recently discovered in unrelated species such as the common swift (*Apus apus*), the lesser short-nosed bat (*Cynopterus brachyotis*) and the buff-tailed bumblebee (*Bombus terrestris*), due to advances in recording capabilities and techniques. They indicate reduced strength of the bound circulation between the wings that results in a lower span efficiency but potentially also a higher degree of manoeuvrability [23]. That even swifts, which nearly spent their entire life on the wing [30] and presumably would benefit from high energy efficiency, exhibit root vortices gives reason to believe that this may be an inevitable feature–at least to some degree–in flying animals. We have demonstrated that paired vortex wakes are time-varying structures and that the location of PIV measurement planes will have an effect on animal flight aerodynamic analyses that should be considered at the stage of experimental design. Because the estimation of lift forces relies on unaltered wakes over the time interval between generation and measurement, consideration is required with respect to positioning of PIV planes and to the theoretical models with which the empirical data are analysed. Of course, the dynamic wakes generated by birds, bats and insects are not identical to the simplified wake topology we produced in this study using fixed wings. Furthermore, root vortices found in the wakes of animals are usually weaker than tip vortices because of the presence of the body and mostly persist only throughout the downstroke, or parts thereof. However, their existence will have an effect on the wake system in the same manner, albeit to a variable extent. As a consequence, force estimates made with the assumption of frozen wakes to date, may contain a source of error that has not been fully acknowledged.

Detailed measurement have been made in the wake of a blackcap *Sylvia atricapilla* L. [17] and of a swift, L. [4]. In both studies root vortices were observed in the flow visualizations. In order to estimate the forces, the former used a vortex ring model to calculate a value for lift that accounted for 99.7±4.7% of the weight of the blackcap. Using a circulation model, the latter study estimated lift only between 56% and 58%–depending on flight speed–of the force required to support the weight of the swift. Although different models were used, a comparison is legitimate since the circulation model can be transformed into the vortex model by integration over the time period of one wing beat (equations 1 and 4). Moreover, the presence of root vortices was accounted for in the same manner; that is, the force was calculated over the span of the tip vortices and, from that value, the force calculated over the span of the root vortices was subtracted. The underestimation of lift force by >40% might, therefore, be due to deformation of the wake. Our results have shown that the parameters with the largest effect are the downstream distance, the gap between wings, and the aspect ratio, but these parameters vary little between the blackcap and the swift and, as such, do not provide an explanation for the force deficit. However, there is a substantial difference in the relative strengths of the root vortices. The circulation in the root vortices recorded behind the swift is half the value of the circulation in the tip vortices, whereas for the blackcap the root to tip circulation ratio is 1∶5. We assume that root vortices as weak as those of the blackcap result in correspondingly reduced interaction. In the case of the swift however, we may assume that wake interaction effects are present, even though they will be less pronounced than in our physical model. A rough check can be made if we simplify and look only at how much the tip vortex wake span of the swift would need to increase in order to achieve full weight support. An increase to 0.24, 0.26 and 0.28 m for the three speeds would be needed compared to 0.14, 0.15 and 0.16 m which were actually measured in the wake. Behind an elliptically loaded wing the wake contracts by a factor of π/4. Although we do not expect the loading on the wings of swift to be perfectly eliptical it would, in this case, result in a wakespan of approximately 0.30 m since the wing span of the swift was 0.38 m. The fact that the measured span is so much less indicates that something else is at play and we propose that part of this is due to wake interaction. For bats, similar force underestimates have been calculated. Bats are now known to produce strong counter rotating vortices near the body [5], [29], [31], which implies significant interaction and, potentially, force deficit.

Due to the conservation of momentum, the wake span behind each wing does not change once the vortex sheet has rolled-up [32]. A possible consequence could be that the tip vortices ‘hinge’ about the root vortices, which brings them closer together and causes an underestimate of the actual span over which force is generated. At the same time, we observed that the distance between root vortices increases during the roll-up of the vortex sheet. This is physically reasonable, since there is no aerodynamic surface generating momentum in the gap and thus the expansion between the root vortices is simply a consequence of the contraction of the wake behind each single wing. The gap between the wings emulates the case of an animal with a body that generates zero lift. In reality, the body of some species does generate lift, so the configuration presented here is a simplification.

Beside the *circulation model* and the *momentum ring model*, one of the first models to investigate flapping flight, the *actuator disc model* [7], has recently regained attention. As with all simplifications, the original form of the model is some way from a realistic representation of the wake since it assumes the generation of a continuous jet flow with an even distribution of induced flows across the disc [33], [34]. Over the last few years, time-resolved PIV measurements have led to refinement of the theory’s application for animal flight, as the downwash velocity, ω, can be acquired along the wingspan at multiple time instances during a stoke period and the momentum is subsequently approximated [2], [5]. Using these data to determine the lift force, only the vertical weight-supporting component of the induced velocity vectors is considered. In light of the vortex interaction in the wake we present here, this approach can lead to incorrect velocity estimates when measuring too far behind the trailing edge due to rotation of the trailing vortices relative to one another. A solution would be to measure immediately behind the trailing edges of the wings, taking into account the safety requirements for PIV experiments with vertebrates [35].

Often, a goal of flapping flight research is to relate time-varying estimates of force generation to the timing of wing stroke kinematics. When measuring in the far wake, deformation of the wake in the manner described here could mislead the observer about the position of the wing at the actual time of force generation. Indeed, the wake deformation as it evolves with downstream spatial displacement we present in Figure 3 closely resembles time-series data from flapping animals. Where deformation is expected to play a significant role, additional cameras that record wing kinematics are important for the correct linkage of wing motion to aerodynamic structures.

### The Mutual Interaction Behaviour of Root Vortices

We observed an interaction of the root vortices with one twisting around the other with downstream displacement (Fig. 3C-E where the second and third vortices rotate around an axis between them). This previously described phenomenon can be found either in vortices with the same strength and the same sense of rotation [28] or between counter-rotating vortices of different strength [36]. In the former case the twisting is a result of induced velocities of opposite directions. In the latter, the vortices affect each other by imposing a straining field on each other but this effect cancels out when the vortices have identical strength – which should be the case for the root vortices in our experiment. Since the opposing wake angle behind each lifting wing tends to force the wakes together, it is inevitable that one root vortex will ultimately push above the other. Which one will rise and which will fall should be random but we found the pattern in Figure 3 to be consistent in our experiments with vortex two rising above vortex three, as shown. The reasons for this remain unknown but it is possible that the freestream retains a residual rotation from the fans (below measurement error with PIV and undetectable with smoke flow visualization) that is sufficient to bias the mutual strain fields of the root vortices. We can eliminate the possibility of undetectable asymmetry in the circulation around each wing (and hence in the trailing vortex strength) due to our repeated measures protocol where the wings were frequently removed and reset on the mounts, and attached to the left or right mounts randomly.

## Concluding Remarks

In this study we have shown that wake topologies that comprise two wing tip and two wing root vortices are not consistent with the assumption of a frozen flow. We found that the wake progressively deforms such that the wingtip vortices convects downwards while the root vortices eventually twisted around each other in the far wake. Wake deformation was strongest behind wings with lower aspect ratios and when the distance between the wings was small. Bearing this in mind, animals with low aspect ratios, petiolated wings and/or where little lift is generated over the body may generate wakes that get particularly deformed. Deformation of the wake can lead to the underestimation of lift when conventional wake models that rely on the assumption of frozen flow are applied.
